# Track-before-Detect Algorithm for Underwater Diver Based on Knowledge-Aided Particle Filter

**DOI:** 10.3390/s22249649

**Published:** 2022-12-09

**Authors:** Wenrong Yue, Feng Xu, Xiongwei Xiao, Juan Yang

**Affiliations:** 1 Ocean Acoustic Technology Laboratory, Institute of Acoustics, Chinese Academy of Sciences, Beijing 100190, China; 2 University of Chinese Academy of Sciences, Beijing 100049, China

**Keywords:** active sonar, track-before-detect, knowledge-aided, particle filter, non-parametric kernel density estimation

## Abstract

This work studies the underwater detection and tracking of diver targets under a low signal-to-reverberation ratio (SRR) in active sonar systems. In particular, a particle filter track-before-detect based on a knowledge-aided (KA-PF-TBD) algorithm is proposed. Specifically, the original echo data is directly used as the input of the algorithm, which avoids the information loss caused by threshold detection. Considering the prior motion knowledge of the underwater diver target, we established a multi-directional motion model as the state transition model. An efficient method for calculating the statistical characteristics of echo data about the extended target is proposed based on the non-parametric kernel density estimation theory. The multi-directional movement model set and the statistical characteristics of the echo data are used as the knowledge-aided information of the particle filter process: this is used to calculate the particle weight with the sub-area instead of the whole area, and then the particles with the highest weight are used to estimate the target state. Finally, the effectiveness of the proposed algorithm is proved by simulation and sea-level experimental data analysis through joint evaluation of detection and tracking performance.

## 1. Introduction

The signal-to-reverberation ratio (SRR) decreases with the increasing complexity of targets and marine environments, resulting in reduced detection performance of sonar equipment. Traditional active sonar regards detection and tracking as two separate subsystems. The detection system sets the threshold using constant false alarm technology to detect the echo signal. If the echo intensity exceeds the threshold, it is considered a target; otherwise, it is regarded as clutter and filtered out. The obtained point-trace information is then passed to the tracking system. When the target motion model is known, some common tracking algorithms estimate the target trajectories using the obtained point trace information. In this method, the accuracy of detection depends on tracking performance. However, in low SRR, the lack of target information reduces tracking accuracy since the threshold filters the weak target. If a lower threshold is used to improve the detection rate of weak targets, it will cause many false alarms. This significantly increases both the difficulty of associating and the computational cost.

The track-before-detect (TBD) methods address the target detection and tracking problem in low SRR [1]. This method accumulates the test statistic according to possible target trajectories, and the threshold decision is then made. Finally, target tracking is realized through traceback. The commonly used TBD algorithms include the dynamic programming TBD algorithms (DP-TBD) [2,3,4,5,6,7,8], Hough transform TBD algorithms (HF-TBD) [9,10,11], and particle filter TBD algorithms (PF-TBD) [12,13,14,15,16,17,18,19,20,21,22,23,24]. The DP-TBD algorithms generally require state space discretization and are very computationally intensive. The HF-TBD algorithms are only suitable for a rectilinear motion target. The PF-TBD algorithms are applied to nonlinear non-Gaussian problems and are more flexible than the former two methods.

At present, the PF-TBD method has been applied in infrared [13,14,15], radar [16,17,18,19], sonar [20,21,22,23,24], and other fields. Most researchers evaluate the PF-TBD method by simulation. However, in [16], Guerraou et al. use the PF-TBD method to detect and track the real target on marine radar. To address weak target detection and tracking in multi-spectral infrared images, a PF-TBD algorithm based on a measurement fusion strategy is proposed by [13]. Bao et al. derive a multi-model optimal particle filter track-before-detect (MMPF-TBD) algorithm for maneuvering weak targets [15]. This algorithm can estimate the target’s state and the existence of the target separately, and improve the particle utilization rate. To solve the problem of target loss or poor tracking accuracy caused by particle impoverishment, Huang et al. propose an improved TBD method that combines the auxiliary particle filter and the multiple-model filter (AUX-MMPF-TBD) [17]. Tian et al. propose a PF-TBD method based on the spring model firefly algorithm, and this method can guide low-weight particles to move in the direction of the high likelihood region, thereby improving particle quality [12]. Awadhiya presents a weight update method based on previous moment feedback for PF-TBD [18]. For an underwater target, Jing combines a standard particle filter with the track-before-detect method to solve the problem of underwater target detection and tracking [20]. Yi proposed a PF-TBD method for passive array sonar target detection [23]. This method minimizes the Cramer-von (CV) distance to obtain the statistical characteristics of the spectrum measurement data.

The TBD method has two key factors: the test statistic and the target motion path. First, a good test statistic can distinguish the target from the clutter. Common test statistics for point targets are amplitude [13], complex amplitude [9], or the likelihood ratio between the power of the target and that of clutter [7]. However, active sonar has the characteristics of high resolution, and the target appears in multi-resolution cells. Although some point spread functions (PSFs) [25] are used to model the energy diffusion over the resolution cells [26], the limited PSFs are too simple to reflect the variations in the characteristics of the target in the sonar system. Secondly, the exact target motion paths on frames are established to accumulate the test statistics in the correct direction. However, whether in single-mode or multi-mode PF-TBD algorithms, the established motion models are mostly uniform motion or uniform turning motion without considering the target motion characteristics in practical applications.

A track-before-detect based on a knowledge-aided particle filter (KA-PF-TBD) algorithm for an underwater diver was proposed. First, based on the motion characteristics of the diver target, a multi-direction motion model set is developed as the target state transition model, which can guide the particle state transition more accurately. To address the problem that the likelihood ratio calculation is inaccurate due to the difficulty in modeling the weak extended underwater target, we use the non-parametric kernel density estimation method to simulate the statistical characteristics of echo data in the sub-area rather than the whole area, which reduces the calculation time with little loss of detection and tracking performance. Therefore, we solve the problem of the underwater diver detecting and tracking by using the multi-directional movement model set of the diver, and the statistical characteristics of the real echo data, as knowledge-aided information in the filtering process. Finally, joint detection and tracking performance indicators are proposed to evaluate the algorithm performance.

The structure of this paper is as follows: In Section 2, the sonar measurement model and target state transition models are given. In Section 3, we develop a KA-PF-TBD method framework for active sonar systems, which includes constructing the diver multi-directional movement model set, and acquiring the measurement data likelihood function and particle filtering process. In Section 4, the effectiveness and efficiency of the KA-PF-TBD method are confirmed both in simulation and in the sea trial data. In Section 5, we summarize the results.

## 2. System Models

### 2.1. Sonar Measurement Model

Assuming that the position of the target $x^{t}$ located in the far field at time t is $x_{p_{t}}$ and $y_{p_{t}}$, and the corresponding velocities are $v_{x_{t}}$ and $v_{y_{t}}$, respectively, then the target state at time t is $x^{t}=\left[x_{p_{t}},v_{x_{t}},y_{p_{t}},v_{y_{t}}\right]$. In an ideal condition without considering noise, the relationship between sonar distance measurement, angle measurement, and target location is:

(1) $$\left\{ \begin{array}{l} r_{t}=\sqrt{x_{p_{t}}^{2}+y_{p_{t}}^{2}}\\ \theta_{t}=\arctan\left(\frac{x_{p_{t}}}{y_{p_{t}}}\right) \end{array}\right.,$$

The sonar system consists of a transmitting array element and a uniform linear array. M is the number of receiving array elements, and d is the array element spacing. If the transmitted signal is s(t), the signal received by M array elements is written as a vector:

(2) $$r\left(t\right)=a\left(\theta_{t}\right)As\left(t-\tau_{tt+tr}\right)e^{jw\left(t-\tau_{tt+rt}\right)}+v\left(t\right),$$

where A is the reflection coefficient of the target; the carrier angular frequency is w; tt stands for signal from transmission to target; tr represents the signal from the target to the receiver; the propagation time delay between transmission and reception is $\tau_{tt+tr}=\frac{2r_{t}}{c}$; and c denotes the speed of sound. The propagation delay is denoted by $\tau_{m}\left(\theta_{t}\right)=\left(m-1\right)d\sin\theta_{t}/c$; $r\left(t\right)={\left[\begin{array}{ccc} r^{1}\left(t\right) & \cdots & r^{m}\left(t\right) \end{array}\right]}^{\prime }$ denotes the vector that is composed of the received signal; $a\left(\theta_{t}\right)={\left[\begin{array}{cccc} 1 & e^{-jw\tau_{1}\left(\theta_{t}\right)} & \cdots & e^{-jw\tau_{m}\left(\theta_{t}\right)} \end{array}\right]}^{\prime }$ denotes the steering vector; and the noise vector is $v\left(t\right)={\left[\begin{array}{ccc} v^{1}\left(t\right) & \cdots & v^{m}\left(t\right) \end{array}\right]}^{\prime }$.

In general, because the complex carrier does not carry any useful information, we only consider complex baseband signals [27]. Thus, the discrete form of (2) is:

(3) $$r\left(k\right)=a\left(\theta_{k}\right)As\left(k-\tau_{tt+tr}\right)+v\left(k\right),$$

where k denotes the *k*-th sample time.

In this paper, after matched filtering and beamforming, the echo signal r(k) is used as the original measurement of the track-before-detect algorithm. Taking the location of the sonar as the origin, the observation area of interest is limited. The distance range is $\left[R_{\min},R_{\max}\right]$, which is divided into $N_{r}$ distance units, and the azimuth range is $\left[\theta_{\min},\theta_{\max}\right]$, which is divided into $N_{b}$ azimuth units, according to Equation (4):

(4) $$N_{r}=\frac{2\left(R_{\max}-R_{\min}\right)}{c}\times F_{s},$$

where $F_{s}$ is the sampling frequency in array signal processing; and $N_{b}$ can be determined by the direction resolution unit, Δθ.

Then the measurement Δθ at time k contains $N_{r}\times N_{b}$ data, which is defined as:

(5) $$z_{k}=\left\{ \begin{array}{cc} g\left(y\left(k\right)\right)+w_{k} & \mathrm{The}\;\mathrm{target}\;\mathrm{exists}.\\ w_{k} & \mathrm{The}\;\mathrm{target}\;\mathrm{does}\;\mathrm{not}\;\mathrm{exist}. \end{array}\right.$$

Among them, g(⋅) is the mapping between signal y(k) and the measurement, and its form affects the specific form of its measurement; and $w_{k}$ is the measurement of noise and clutter of the system at time, k.

### 2.2. Target State Transition Model

If the target state transition model matches the actual target motion, the filter has good tracking performance. If the filter fails to track, it is important for the tracking system to establish an appropriate target state transition model. The general expression for target state transition is:

(6) $$x_{k+1}=f\left(x_{k},\tau_{k}\right)+v_{k},$$

where the target state is denoted by $x_{k}$; $\tau_{k}$ represents the target motion model type; $v_{k}$ is the corresponding process noise; and f(⋅) is the state transition matrix under different models.

In this paper, $\tau_{k}=1$ and $\tau_{k}=2$ are the commonly used uniform and cooperative turning motion models, respectively, and $w_{k}$ is the cooperative turning rate.

(7) $$f\left(x_{k},\tau_{k}=1\right)=\left[\begin{array}{cccc} 1 & T & 0 & 0\\ 0 & 1 & 0 & 0\\ 0 & 0 & 1 & T\\ 0 & 0 & 0 & 1 \end{array}\right],$$

(8) $$f\left(x_{k},\tau_{k}=2\right)=\left[\begin{array}{cccc} 1 & \frac{\sin\left(w_{k}T\right)}{w_{k}} & \frac{\cos\left(w_{k}T\right)-1}{w_{k}} & 0\\ 0 & \cos\left(w_{k}T\right) & 0 & \sin\left(w_{k}T\right)\\ 0 & \frac{1-\cos\left(w_{k}T\right)}{w_{k}} & 1 & \frac{\sin\left(w_{k}T\right)}{w_{k}}\\ 0 & \sin\left(w_{k}T\right) & 0 & \cos\left(w_{k}T\right) \end{array}\right].$$

The transition probability between multiple models at time k can be represented by a Markov chain:

(9) $$P\left\{\tau_{k}=j|\tau_{k-1}=i\right\}=p_{ij},i,j=1,2.$$

## 3. Algorithm Development

**Figure 1:**
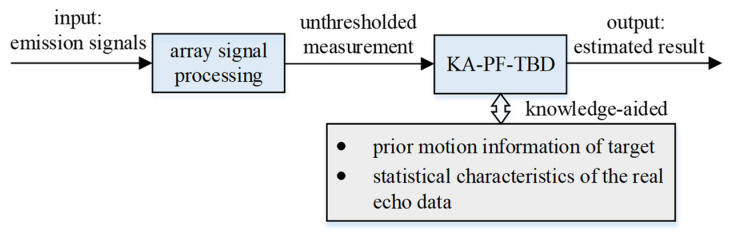
The KA-PF-TBD signal processing procedure flowchart.

We propose a KA-PF-TBD method for underwater diver target tracking in active sonar systems. Figure 1 shows the flowchart of this method, and as shown, the input of the method is the unthresholded measurements, and the output of the method is the target tracking results. The important steps include the construction of the diver multi-directional movement model set, the acquisition of the measurement data likelihood function, and the particle filtering process.

### 3.1. The Construction of the Diver Multi-Directional Movement Model Set

**Figure 2:**
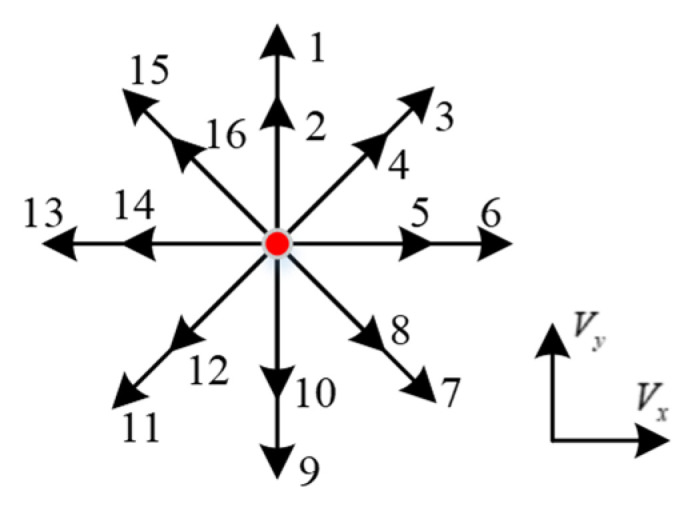
The diver multidirectional movement model set.

Compared with the maneuvering target, the diver target has its own unique motion characteristics. It is difficult for its motion direction to be predicted, and there is little change in the speed of motion between adjacent moments. To describe the low-speed and high-directional change rate of the diver, as shown in Figure 2, a multi-directional motion model set composed of 8 directions and 16 uniform linear motions is established in this paper.

In Figure 2, the red circle represents the position of the diver target at the current moment, and the direction indicated by the eight arrows is the possible predicted direction of the target. Each direction includes two uniform linear motion models with speeds of 0.75 and 1.25 times the target speed range at the previous moment. $V_{x}$ and $V_{y}$ denote the velocity direction of the target state estimation at the current time.

### 3.2. The Acquisition of the Measurement Data Likelihood Function

The non-parametric kernel density estimation does not make any assumptions about the distribution of measurement data, and only models the probability density function based on the sample data itself. This method is frequently used in financial risk prediction and estimation [28], industrial machinery residual life prediction and estimation [29], and so on. This paper uses the non-parametric kernel density estimation theory to fit the statistical properties of sonar measurement data.

Assuming that $z_{1},z_{2},\cdots ,z_{n}$ are the n measurement data samples, and $\hat{g}\left(z\right)$ is the kernel density estimation of the sample probability density function, then the expression of $\hat{g}\left(z\right)$ is:

(10) $$\hat{g}\left(z\right)=\frac{1}{nh}\sum_{i=1}^{n}K\left(\frac{z-z_{i}}{h}\right),$$

where n is the number of independent identically distributed samples; K(⋅) represents the kernel function, which determines the role of each sample data point $z_{i},i=1,\cdots ,n$ in the density estimation of random variable z; and h is the window width that affects the smoothness of the probability density estimation.

After obtaining the statistical characteristics of real echo data, the likelihood function of the measurement resolution unit (i,j) can be calculated from:

(11) $$l\left(z_{k}^{\left(i,j\right)}|x_{k}^{},E_{k}^{}\right)=\left\{ \begin{array}{ll} \frac{g_{1}\left(z_{k}^{\left(i,j\right)}|x_{k}^{},E_{k}^{}=1\right)}{g_{0}\left(z_{k}^{\left(i,j\right)}|E_{k}^{}=0\right)} & E_{k}^{}=1\\ 1 & E_{k}^{}=0 \end{array}\right.,$$

where $g_{1}\left(\cdot \right)$ denotes the statistical property of measurement data when the target exists, and $g_{0}\left(\cdot \right)$ denotes the statistical property of measurement data when the target does not exist. Since the target occupies multiple resolution units in the measurement space, the likelihood function is expressed as,

(12) $$l\left(z_{k}|x_{k}^{},E_{k}^{}\right)=\prod_{i,j\in \Theta }l\left(z_{k}^{\left(i,j\right)}|x_{k}^{},E_{k}^{}\right),$$

where Θ is the range of target influence resolution units, which will be detailed in Section 4.

### 3.3. Algorithm Steps

**Figure 3:**
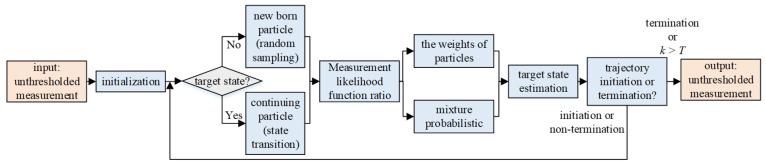
Iteration steps.

If the particle set ${\left\{x_{k-1}^{A,i},w_{k-1}^{i}\right\}}_{i=1}^{N_{c}}$ at time k−1 can be used to describe the posterior probability density $p\left(x_{k-1}^{A},E_{k-1}|z_{1:k-1}\right)$, then one iteration of the algorithm is shown in Figure 3.

Steps 1: At the initial moment, only newly born particles are generated. If the prior distribution of the target is known, the particle is generated according to its distribution; if there is no prior information on the target, the samples are uniformly sampled in the observation area.

Steps 2: According to the prior probability distribution, $N_{b}^{}$ new-born particles are generated; using the established diver motion model as knowledge-aided information, the state of the $N_{c}$ continuing particles is estimated as follows:

(13) $$x_{k}^{\left(b\right)i}∼q\left(x_{k}|E_{k}=1,E_{k-1}=0,z_{k}\right).$$

(14) $$x_{k}^{\left(c\right)i}∼q\left(x_{k}|x_{k-1},E_{k}=1,E_{k-1}=1,z_{k}\right).$$

Steps 3: The statistical characteristics of measurement in the previous section are used as knowledge-aided information. The weight of new particles and continuing particles are calculated and normalized as follows.

(15) $${\tilde{w}}_{k}^{\left(b\right)i}=\frac{l\left(z_{k}|x_{k}^{\left(b\right)i},E_{k}^{\left(b\right)i}=1\right)p\left(x_{k}^{\left(b\right)i}|E_{k}^{\left(b\right)i}=1,E_{k-1}^{\left(b\right)i}=0\right)}{N_{b}q\left(x_{k}^{\left(b\right)i}|E_{k}^{\left(b\right)i}=1,E_{k-1}^{\left(b\right)i}=0,z_{k}\right)}.$$

(16) $${\tilde{w}}_{k}^{\left(c\right)i}=\frac{l\left(z_{k}|x_{k}^{\left(c\right)i},E_{k}^{\left(b\right)i}=1\right)}{N_{c}}.$$

(17) $$w_{k}^{\left(b\right)i}=\frac{{\tilde{w}}_{k}^{\left(b\right)i}}{\sum_{i=1}^{N_{b}}{\tilde{w}}_{k}^{\left(b\right)i}}.$$

(18) $$w_{k}^{\left(c\right)i}=\frac{{\tilde{w}}_{k}^{\left(c\right)i}}{\sum_{i=1}^{N_{c}}{\tilde{w}}_{k}^{\left(c\right)i}}.$$

Steps 4: Non-normalized weights are used to calculate the mixing probability and then it is normalized.

(19) $${\tilde{M}}_{b}=P_{b}\left(1-{\hat{P}}_{k-1}\right)\sum_{i=1}^{N_{b}}{\tilde{w}}_{k}^{\left(b\right)i}.$$

(20) $${\tilde{M}}_{c}=\left(1-P_{d}\right){\hat{P}}_{k-1}\sum_{i=1}^{N_{c}}{\tilde{w}}_{k}^{\left(c\right)i}.$$

(21) $$M_{b}=\frac{{\tilde{M}}_{b}}{{\tilde{M}}_{b}+{\tilde{M}}_{c}}.$$

(22) $$M_{c}=\frac{{\tilde{M}}_{c}}{{\tilde{M}}_{b}+{\tilde{M}}_{c}}.$$

Steps 5: The weight of new born and continuing particle is scaled according to the mixing probability.

(23) $${\overset{⌢}{w}}_{k}^{\left(b\right)i}=M_{b}w_{k}^{\left(b\right)i}.$$

(24) $${\overset{⌢}{w}}_{k}^{\left(c\right)i}=M_{c}w_{k}^{\left(c\right)i}.$$

Steps 6: The new and continuing particles form a complete particle set. They are resampled to obtain ${\left\{x_{k}^{A,i}\right\}}_{i=1}^{N_{c}}$, and then the target state ${\left\{x_{k}^{A,i}\right\}}_{i=1}^{N_{c}}$ is estimated. In the next simulation experiments, we use the system resampling method.

(25) $$\left\{\left(x_{k}^{\left(t\right)i},{\overset{⌢}{w}}_{k}^{\left(t\right)i}\right)|i=1,\cdots ,N_{t},t=b,c\right\}.$$

(26) $${\hat{x}}_{k}=\frac{\sum_{i=1}^{N_{c}}x_{k}^{i}}{N_{c}}.$$

Steps 7: In this algorithm, target detection and tracking are realized by particles, and the initiation and termination of trajectories are actually related to the initiation and termination of particles. The particles are generated by importance sampling, so the importance density can be designed to determine the trajectory initiation and termination.

According to reference [21], for each target with $N_{t}$ frames, the $\Sigma_{w}$ sum of all particles weight is calculated.

(27) $$\Sigma_{w}=\sum_{j=1}^{N_{t}}\sum_{i=1}^{N_{b}+N_{c}}{\tilde{w}}_{j}^{i}.$$

If:

(28) $$\Sigma_{w}<\eta_{d},$$

then the target initiation fails or terminates, and $\eta_{d}$ is the likelihood threshold.

Steps 8: The target track results are generated as output.

## 4. Numerical Results

In this section, the performance of the proposed KA-PF-TBD method is evaluated by analyzing the simulated data and the trial data which are compared with the MMPF-TBD [15] and the AUX-MMPF-TBD [17]. The experiments are performed on our computer using an Intel i5-12500H CPU (2.50 G) and 16 GB memory.

### 4.1. Evaluation Indicators

Evaluating a track-before-detect algorithm is itself a challenge. A common quantitative evaluation indicator is the position’s root mean square error. However, this indicator does not always accurately measure tracking performance. For instance, if an algorithm tracks a target properly in most cases but fails to do so in a few cases, the mean error may be higher than that generated by the algorithm without accurate tracking. In addition, the indicator only evaluates the tracking performance without considering the detection performance. For the above reasons, we also use the following indicators in addition to the position’s root mean square error.

The position’s root mean square error (RMSE): This is used to evaluate the tracking performance well, and it is defined as

(29) $$RMSE=\sqrt{\sum_{i=1}^{m}\left[{\left({\hat{x}}_{k}^{i}-x_{k}\right)}^{2}+{\left({\hat{y}}_{k}^{i}-y_{k}\right)}^{2}\right]/m},$$

where m is the Monte Carlo (MC) experiment times.

The accurate detection probability sequence $P_{d}$: This consists of the *N*-frame accuracy detection probability of the target. The single-frame accurate detection probability is the probability that the frame is accurately detected in multiple MC simulations. The steeper the rising edge of the accurate detection probability sequence curve is, the faster is the effective track formed. The accurate detection probability sequence curve tends to be stable, and the smaller the fluctuation is, the stronger the robustness of the algorithm.

The stable detection and tracking probability $P_{dt}$: In the MC experiments, if the proportion of the number of traces of the effective track in the total number of traces exceeds the predetermined proportion, the experiment is said to have achieved stable detection and tracking of the target. The proportion of the number of experiments to achieve stable detection and tracking relative to the total number of experiments is called the stable detection and tracking probability. Compared with the traditional single-frame detection probability, this indicator no longer considers the plot alone but also the probability of stable detection and tracking of the target based on the effective track. It is a joint evaluation of detection and tracking performance.

The precision plot: This plot shows the percentage of frames for which the estimated object location was within some threshold distance of the actual position. If the algorithm has higher tracking accuracy at a lower threshold, it can achieve more accurate target tracking.

### 4.2. Simulation Experiments

In this section, to test the performance of the proposed KA-PF-TBD method, we designed a simulation in which the diver target makes a compound movement in the plane. For our purposes, we make the assumption that the target shows up at 10 s and disappears at 120 s. The initial state is set as follows:

(30) $$x_{0}=\left[100,0.5,150,0.5\right].$$

**Figure 4:**
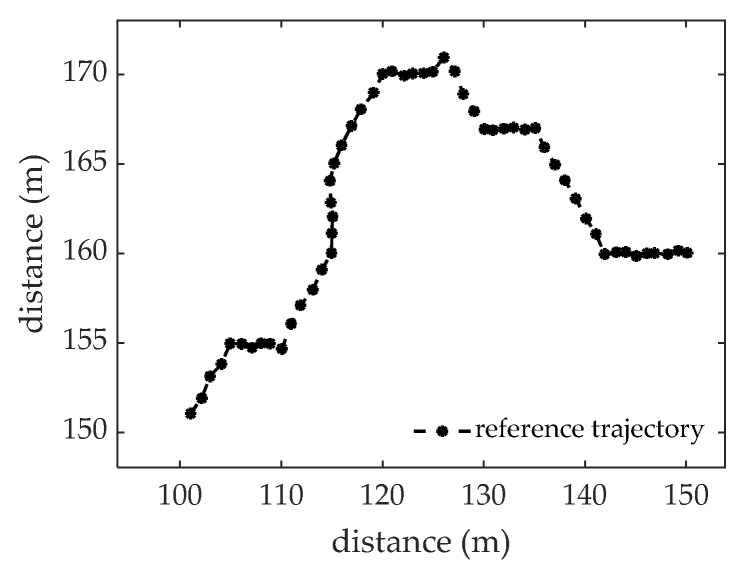
The reference trajectory.

The reference trajectory is shown in Figure 4.

The sonar emits linear frequency modulation (LFM) signals and the sampling interval is T=2 s. The measurement range is $R_{\min}=150\;m$ to $R_{\max}=250\;m$; and angle range is $\theta_{\min}=20^{\circ }$, $\theta_{\max}=80^{\circ }$. Taking SRR = 10 dB and SRR = −5 dB as examples, the non-parametric kernel density estimation method is used to simulate the statistical characteristics of the echo data. The effect of the size of the sub-area on the performance of the algorithm—and how to select the appropriate sub-area through the tradeoff between efficiency and accuracy—are explained.

**Figure 5:**
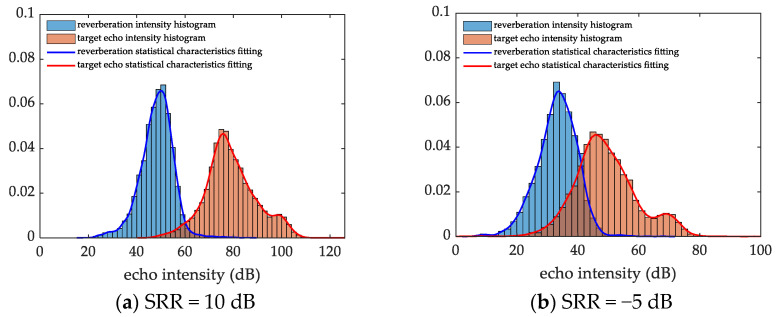
Statistical characteristics of reverberation and target echo data, showing the fitting results when: (**a**) the SRR is 10 dB. (**b**) the SRR is −5 dB.

Figure 5a,b displays the fitting results of the statistical characteristics of the echo data when the SRR is 10 dB and −5 dB, respectively. It can be seen from Figure 5 that the smaller the SRR is, the larger the overlap between the noise and the target statistical characteristics, and the more difficult it is to distinguish the target from the clutter.

The six setups represent six different sizes of the sub-area. The stable detection and tracking probability, the position’s root mean square error, and the computation time are compared in Table 1.

It can be seen from Table 1 that using a sub-area instead of the whole area to calculate the likelihood ratio can greatly improve computational efficiency. The smaller the size of the sub-area, the less computation time is required. However, the size of the subarea affects the accuracy of the algorithm. In all settings, setup 1, 2, and 3 have higher computational efficiency, but they have obvious performance degradation. Although the detection and tracking performance of setup 5 and 6 are good, the computational efficiency is low, and cannot meet the real-time requirements. Compared to other setups, setup 4 has higher computational efficiency with little loss of accuracy, so in the subsequent experiments, the likelihood ratio is calculated on the basis of setup 4 instead of the whole area.

To ensure the fairness of the algorithm comparison, the three methods keep the same parameter settings. The number of particles is 1500, the birth probability of particles is $P_{b}=0.85$, and the death probability of particles is $P_{d}=0.15$. Birth information is an a priori for target births, similar to [30].

$$\begin{array}{l} x_{p_{0}}\sim U\left[90,160\right]\\ y_{p_{0}}\sim U\left[140,170\right]\\ v_{x_{0}}\sim U\left[-1,1\right]\\ v_{y_{0}}\sim U\left[-1,1\right] \end{array}$$

The transition probability matrix of the commonly used model sets is:

$$P=\left[\begin{array}{cc} 0.85 & 0.15\\ 0.15 & 0.85 \end{array}\right].$$

The transition probability matrix of the multi-directional diver motion model set is:

$$P={\left[\begin{array}{cccc} 0.85 & 0.01 & \cdots & 0.01\\ 0.01 & 0.85 & \cdots & 0.01\\ \vdots & \vdots & \ddots & \vdots \\ 0.01 & \cdots & 0.01 & 0.85 \end{array}\right]}_{16\times 16}.$$

**Figure 6:**
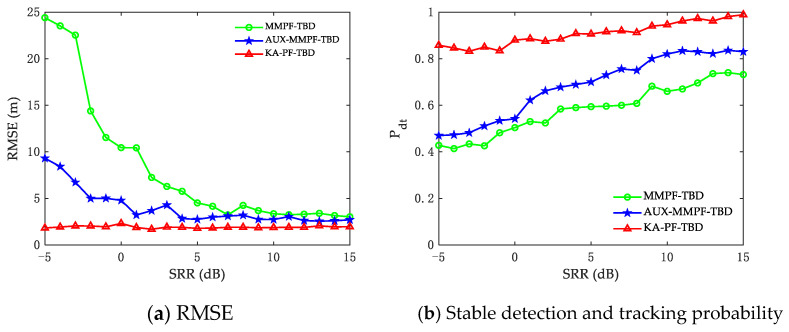
(**a**) The RMSE under different SRR. (**b**) The stable detection and tracking probability under different SRR.

To ensure the reliability of the results, we carried out 100 MC simulation experiments. Figure 6a,b shows that the stable detection and tracking probability of the three methods decrease with the decrease in SRR. The MMPF-TBD algorithm suffers from poor detection and tracking performance when the SRR is lower than 5 dB. Because it uses two conventional state transition models to limit the direction of particle transfer, the prediction results are inconsistent with the actual diver motion state. The performance of AUX-MMPF-TBD is slightly better than that of the MMPF-TBD algorithm because the former uses auxiliary particles to improve particle utilization. Compared with the former two methods, the KA-PF-TBD algorithm has better detection and tracking performance when the target SRR is low. The reason for the performance improvement is that it uses the original measurement as its input and combines the motion characteristics of the diver target to provide the necessary knowledge assistance for detection and tracking.

**Figure 7:**
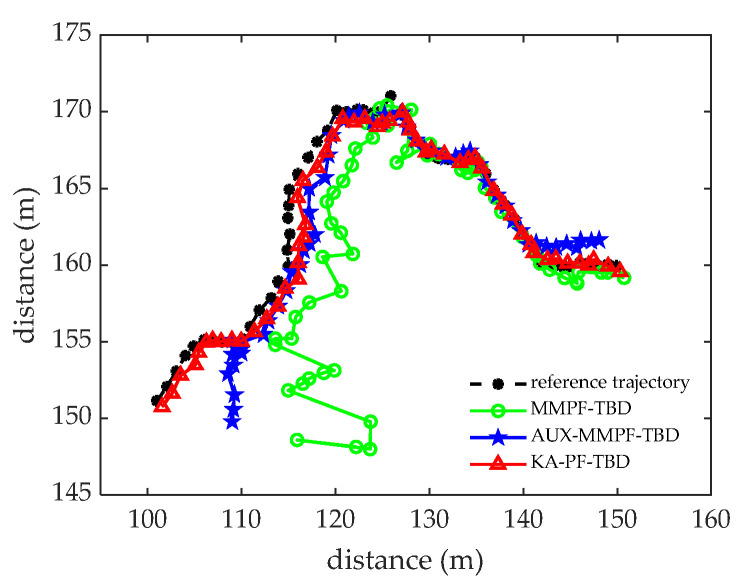
The tracking results.

Figure 7 shows the reference and tracking trajectories obtained using the three algorithms when SRR = 4 dB. In the low SRR scene with an uncertain target direction, the AUX-MMPF-TBD and KA-PF-TBD algorithms can accurately estimate the target direction and position. In contrast, the MMPF-TBD algorithm performs poorly and only tracks the target in a few frames. The first two methods make particles move toward the high likelihood ratio region by using auxiliary particles and improving the target state transition model, so their performance is improved. Since both of them are tracking before the detection algorithms come into effect, the detection decision is made after tracking the target, and the tracking will affect the subsequent detection results.

**Figure 8:**
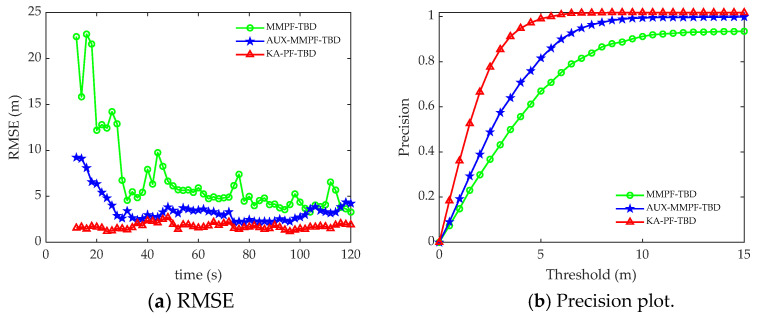
Simulation results. (**a**) The RMSE plotted against time. (**b**) Precision plotted against time.

Figure 8a compares the position RMSE results of the three algorithms under each frame. The position RMSE of the KA-PF-TBD algorithm is much smaller than that of the other two algorithms, and the fluctuation is not large. The tracking accuracy plot of the three methods is shown in Figure 8b. This shows that the KA-PF-TBD method can obtain higher accuracy at a lower threshold, so its tracking is more accurate.

**Figure 9:**
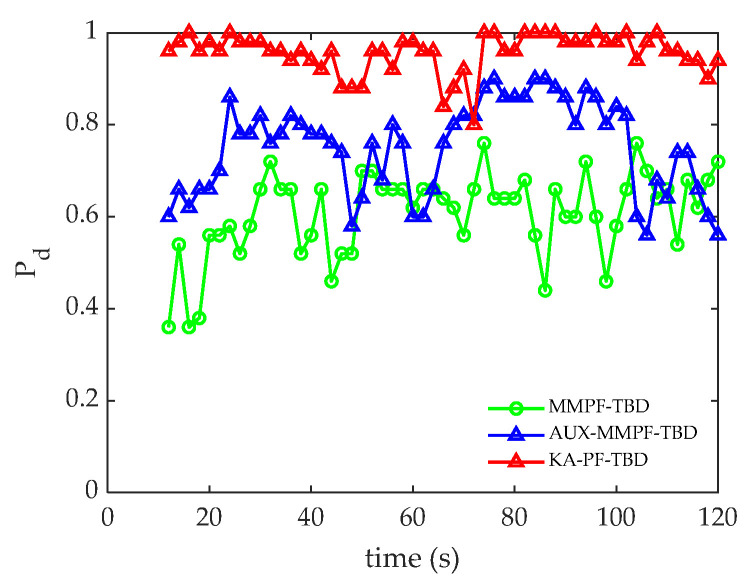
The accurate detection of probability sequences.

Figure 9 presents the accurate detection probability sequence of the three methods. Compared with the MMPF-TBD and AUX-PF-TBD methods, the KA-PF-TBD method has a higher detection probability, which is consistent with the corresponding tracking results. Moreover, because the accurate detection probability curve of the KA-PF-TBD algorithm fluctuates less, the algorithm is more robust.

### 4.3. Sea Trial Experimental Data Processing

**Figure 10:**
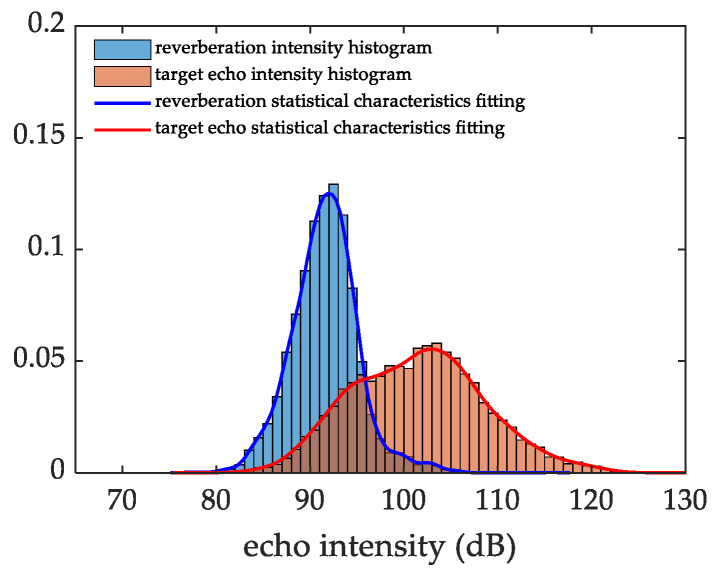
Statistical characteristics of reverberation and target echo data.

In this section, the performance of the KA-PF-TBD method is further confirmed using a set of sea trial experimental data. We collected the trail data in a shallow sea, and recorded the actual position of the diver target using GPS devices. The voyage with reverberation around the target is chosen for data processing to test the effectiveness of the proposed algorithm. First, the non-parametric kernel density estimation method is used to fit the statistical characteristics of the measured data. Figure 10 shows the histogram of the reverberation and target echo data and the fitting results. As shown in the figure, the reverberation and target statistical characteristics largely overlap, which proves that the SRR is low.

**Figure 11:**
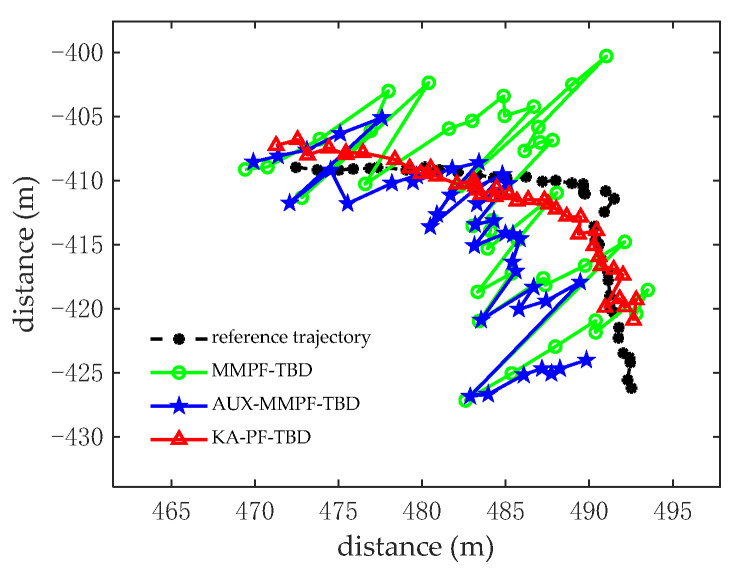
The tracking results.

We then carried out 100 MC simulation experiments. The number of particles used in both algorithms was 3000, the probability of particle birth was $P_{b}=0.85$, and the probability of particle death was $P_{d}=0.15$. Figure 11 shows the tracking results of the three methods. Compared with the MMPF-TBD and AUX-PF-TBD methods, the KA-PF-TBD algorithm estimates the target state more accurately. Because the latter establishes a multi-directional motion model based on the motion characteristics of the diver, the surviving particles can transfer to all directions of the target motion; that is, the particles that predict the target state of the next frame will appear in multiple directions. We then calculated the particle likelihood ratio according to the measurement statistical characteristics of the original data. Finally, the particles with the high likelihood ratio from resampling were selected to estimate the target state.

**Figure 12:**
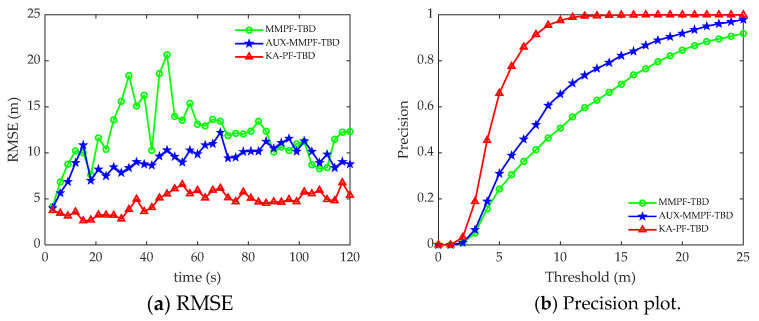
The trial data processing results. (**a**) The RMSE plotted against time. (**b**) Precision plotted against time.

**Figure 13:**
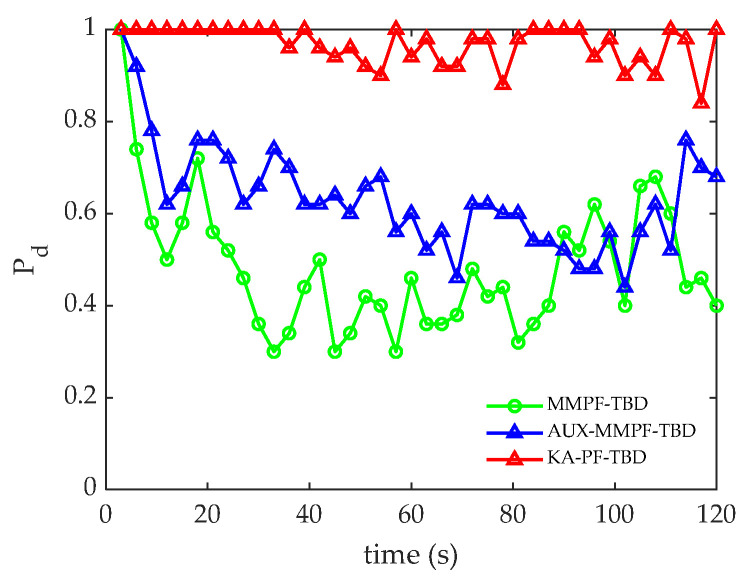
Accurate detection of probability sequences.

Figure 12a,b shows the root mean square error and accuracy curves of the three algorithms at each time. Figure 13 shows the accurate detection probability sequence of the three algorithms at each time, indicating that the proposed KA-PF-TBD method can achieve weak target detection and can track more accurately than the other two methods.

## 5. Conclusions

This work considers underwater diver target tracking in active sonar systems with low signal-to-reverberation (SRR). We proposed a particle filter track-before-detect based on a knowledge-aided (KA-PF-TBD) algorithm to enhance the tracking performance of low SRR diver targets. This method establishes a diver multi-directional motion model set for an underwater diver by making full use of the prior information about the diver target, and solves the problem that the conventional model set does not match the actual target motion. The received original measurement data is directly used as the input of the KA-PF-TBD method to avoid the loss of the target due to threshold processing. We adopt the non-parametric kernel density to simulate the statistical characteristics of echo data, which is used to calculate the particle likelihood ratio with the sub-area instead of the whole area. The effectiveness of the KA-PF-TBD method was verified by simulation and sea trial data processing. Compared with the MMPF-TBD and AUX-MMPF-TBD algorithms, the proposed method detects the diver target with a detection probability higher than 90 % in the sea trial data with a low SRR.

The proposed method in this paper only models the motion state of the diver, but is difficult to apply to all underwater targets due to the diversity of underwater target motion types. The kernelized correlation filter tracker does not depend on any predefined target state transition model [30,31]. Thus, it can obtain the maximum test statistics with a fast and exhaustive search, and track targets with multiple motion types. In addition, extracting the image features or signal features of the target can improve the target recognition ability [32,33]. We will extend the kernelized correlation filter theory-based track-before-detect methods and consider assistance based on feature knowledge in the future.

## Figures and Tables

**Table 1:** Simulation results of the different setups.

	Setup 1	Setup 2	Setup 3	Setup 4	Setup 5	Setup 6
$N_{wr}\times N_{wa}$	2×2	5×5	10×5	20×5	50×10	all
$P_{dt}^{\mathrm{SNR}=10}$	0	0.798	0.79	0.902	0.934	1
$RMSE^{\mathrm{SNR}=10}$		2.075 m	2.11 m	1.696 m	1.331 m	0.19 m
$t_{}^{\mathrm{SNR}=10}$	46.3 s	52.1 s	54.2 s	56.6 s	90.4 s	1805.3 s
